# Multiscale Models of CVD Process: Review and Prospective

**DOI:** 10.3390/ma17205131

**Published:** 2024-10-21

**Authors:** Yu Tian, Zefan Yan, Lin Jiang, Rongzheng Liu, Bing Liu, Youlin Shao, Xu Yang, Malin Liu

**Affiliations:** 1 Institute of Nuclear and New Energy Technology, Tsinghua University, Beijing 100084, China; 2 Hunan Valin Xiangtan Iron and Steel Co., Ltd., Xiangtan 411101, China

**Keywords:** CVD, mechanism, numerical simulation, multiscale, inter-scale coupling

## Abstract

Chemical vapor deposition (CVD) is a crucial technique in the preparation of high-quality thin films and coatings, and is widely used in various industries including semiconductor, optics, and nuclear fuel, due to its operation simplicity and high growth rate. The complexity of the CVD process arises from numerous parameters, such as precursor chemistry, temperature, pressure, gas flow dynamics, and substrate characteristics. These multiscale parameters make the optimization of the CVD process a challenging task. Numerical simulations are widely used to model and analyze the CVD complex systems, and can be divided into nanoscale, mesoscale, and macroscale methods. Numerical simulation is aimed at optimizing the CVD process, but the inter-scale parameters still need to be extracted in modeling processes. However, multiscale coupling modeling becomes a powerful method to solve these challenges by providing a comprehensive framework that integrates phenomena occurring at different scales. This review presents an overview of the CVD process, the common critical parameters, and an in-depth analysis of CVD models in different scales. Then various multiscale models are discussed. This review highlights the models in different scales, integrates these models into multiscale frameworks, discusses typical multiscale coupling CVD models applied in practice, and summarizes the parameters that can transfer information between different scales. Finally, the schemes of multiscale coupling are given as a prospective view. By offering a comprehensive view of the current state of multiscale CVD models, this review aims to bridge the gap between theory and practice, and provide insights that could lead to a more efficient and precise control of the CVD process.

## 1. Introduction

Chemical vapor deposition (CVD) is a widely used technology to prepare thin films and coatings, playing a critical role in industries, including semiconductor, optics, and nuclear fuel. The CVD process involves the chemical reactions of volatile precursor gases on the surface of substrates, leading to the deposition of solid materials [1]. This is the most significant difference between CVD and physical vapor deposition (PVD). CVD is known for its advantages, such as simplicity in operation, efficiency, high quality, wide selections of precursors and substrates, and rapid growth rates [2].

CVD was first used to produce lamp filaments in 19th century [3], and the development of CVD technology was accelerated by the increasing demand for semiconductor thin films after World War II [4,5]. Nowadays, CVD is becoming more popular in the preparations of low-defect density and large-area 2D materials with precise control over layer number, domain size, and morphology [2], such as graphene [6], carbon nanotubes [7], diamond [8], semiconductors [9], photoelectric [10], and nanomaterials [11].

The CVD process involves chemical reactions, and the nucleation and growth of thin films. Despite its extensive application, it is still a complex challenge to optimize the CVD process due to the effects of numerous parameters, including precursor chemistry, temperature, pressure, gas flow dynamics, substrate properties, and time [12]. These parameters interact across multiple scales, from atomistic and molecular levels to reactor scale, thus requiring a comprehensive understanding to achieve precise control over the whole CVD process.

To address these complexities, numerical simulation methods are good choices. Numerical simulation applies a mathematical model to describe a physical system. With the development of computer hardware, numerical simulations have become more popular due to their efficiency and accuracy. These simulations are essential for studying the systems with complex mathematical models, especially in nonlinear systems.

According to the scale of the study object in the CVD process, numerical simulation methods are divided into nanoscale, mesoscale, and macroscale. Density functional theory (DFT) and molecular dynamics (MD) are two typical nanoscale simulation methods. The phase-field model (PF) and kinetic Monte Carlo method (KMC) are commonly used in mesoscale simulations. Computational fluid dynamics (CFD) is the most common macroscale method, and can be divided into Euler–Euler method and Euler–Lagrange method.

The wide range of spatial and temporal scales involved in the CVD process add difficulties to numerical simulations. For instance, DFT is used to calculate the activation energies during chemical reactions and adsorption energies on the substrates [13,14], but is unable to address the phenomena in reactor scale. Therefore, multiscale modeling has become a powerful approach, offering insights that cover different length and time scales. By coupling models that capture phenomena at nanoscopic, mesoscopic, and macroscopic levels, multiscale CVD models provide a more comprehensive understanding of the CVD process. These multiscale models are essential for predicting film growth, optimizing process parameters, and finally improving the film quality and uniformity.

This review aims to provide a comprehensive overview of CVD models, focusing on key steps of the CVD process, critical parameters that influence the CVD process, various models used at different scales, and multiscale CVD models with their applications. This review will bridge the gap between theory and practice, offer valuable insights into the current state of multiscale CVD models, and summarize the parameters that can transfer information between different scales. It will be beneficial for the CVD research community, and also develops the research field of multiscale research methodology.

## 2. Mechanism in the CVD Process

CVD technology has been widely used to prepare high-quality, thin-film, and solid materials. There are many types of CVD process, such as low-pressure CVD (LPCVD), atmospheric-pressure CVD (APCVD), and plasma-enhanced CVD (PECVD).

CVD involves gaseous precursors reacting in the vapor phase or on the substrate, leading to the formation of solid products. Taking graphene as an example, during the preparation of this famous 2D material in the laboratory, Cu substrate is first chosen and cleaned, heated to the specified temperature, and exposed to methane and hydrogen. Under the catalysis of Cu substrate, the methane decomposes into C_x_H_y_ on the Cu substrate. The saturation of C_x_H_y_ species on the Cu surface is dependent on the temperature and pressure, and the nuclei will form because of the local supersaturation of C_x_H_y_. After the growth of nuclei, graphene layers form. Finally, the substrate is cooled under a proper atmosphere to prevent oxidation, and volatile byproducts are removed [1,6,15].

From a more mechanical point of view, CVD has several elementary processes, and can be divided into chemical reactions, adsorption, diffusion, nucleation, growth, and byproduct removal [16].

### 2.1. Chemical Reactions

CVD and PVD are significantly different because the CVD process is accompanied by chemical reactions. It plays an important role in determining film growth rate, deposit composition, and reactor behaviors [17], thus allowing for tunable deposition rates and high-quality products with excellent conformality [18]. Before the CVD process, the reactor and the substrate are heated. Then the precursors undergo reactions in the gas phase or on the substrate surface. Therefore, the reactions can be divided into homogeneous gas-phase reactions and heterogeneous surface reactions.

Heterogeneous reaction occurs at the interface between two different phases, usually between the gas phase and the substrate. A heterogeneous reaction is a fundamental reaction type in CVD. Before the formation of solid film, the gas reactants may undergo various surface processes. This mechanism allows for precise control over the composition and thickness of the products, making it a promising application in the field of semiconductors.

A homogeneous reaction only occurs in the gas phase. In the CVD process, before reaching the substrate, the reactants interact with each other in homogeneous reactions. These reactions lead to the formation of precursors or fine particles that contribute to the deposition process. However, homogeneous reactions may also lead to nucleation in the gas phase, making it difficult to obtain smooth films. Therefore, to ensure high-quality film growth and to minimize defects, homogeneous reactions should be controlled [19].

Also, the CVD process can be divided into non-catalytic and catalytic CVD based on if there are catalysts involved in the chemical reactions. In a non-catalytic CVD process, the chemical reaction is controlled by temperature generally, so the deposition is non-directional [20]. But in the CVD processes with catalysts, the chemical reactions are controlled by the catalytic activity, and the deposition direction can be adjusted [21]. In general, the CVD model is easier to establish for simulating the non-catalytic CVD process, because the catalytical process is much more difficult to describe mathematically.

### 2.2. Adsorption, Diffusion, Nucleation, and Growth

Adsorption refers to the process where atoms or molecules from the gas phase stick to the surface. In CVD, adsorption is a critical step where the precursors interact with the substrate, involving the formation of chemical bonds between the adsorbate and the substrate. The precursor atoms or molecules diffuse through the gas phase toward the substrate, and are influenced by the flow dynamics, pressure, and the temperature in the reactor. In addition, the nature of the substrate, such as its surface energies and temperature, will affect the adsorption process.

After adsorption, the adsorbed atoms may migrate across the surface of the substrate. Surface diffusion is an essential step for atoms to find energetically favorable sites, thus growing uniformly [22], determining the morphology and crystallinity of the films. Atoms or molecules on the substrate can move via different mechanisms, including hopping [23,24], exchange [25,26], and evaporation–condensation [27]. The diffusion rate is quantified by the surface diffusion coefficient D_s_, which can be expressed as:

(1) $$D_{s}=D_{0}\exp\left(\frac{E_{d}}{k_{B}T}\right).$$

where D_0_ is the pre-exponential factor related to the effective hopping distance between sites, E_d_ is the potential-energy barrier from site-to-site. D_s_ determines the average distance that an adatom have to travel before nucleation [28].

Nucleation refers to the initial formation of stable clusters or nuclei of atoms or molecules on the substrate surface, serving as the foundation for film growth. Similar to chemical reactions, the nucleation process can be divided into homogeneous nucleation and heterogeneous nucleation [29]. Homogeneous nucleation occurs in the gas phase without preferential nucleation sites. Heterogeneous nucleation occurs on the surface of the substrate, which lowers the energy barrier for nucleation, making it more favorable than homogeneous nucleation [30]. Surface diffusion allows adatoms to find each other and form clusters. Once reaching the critical size, the clusters will become stable nuclei. The nucleation process determines the density, size, and distribution of clusters or nuclei, thus influencing the film’s overall quality [31].

When nucleation continues, the growth starts, thus forming a thin film. The growth process can occur through various mechanisms, which are relying on the substrate temperature and precursor properties. Layer-by-layer growth [32,33], island growth [34,35], layer-plus-island growth [36], and epitaxial growth [37,38] are common mechanisms. To achieve the desired film characteristics, there are several challenges during the growth process, such as defect formation, uniformity, and grain size.

For different reaction systems, different CVD reactors or process parameters can be chosen. The presence or absence of each elementary step can result in significant changes in the overall process [16].

### 2.3. Byproduct Removal

The chemical reactions in CVD not only result in the desired thin film, but also generate various byproducts. These byproducts can be gases, liquids, or solids and must be removed efficiently to maintain the quality of the thin films. Byproducts in CVD arise from several sources like decomposition of precursor gases, side reactions, incomplete reactions [39,40,41], or surface reactions. Several techniques are employed to remove byproducts from CVD system, such as exhaust and pumping systems, scrubbers, and plasma cleaning [42,43,44].

**Figure 1:**
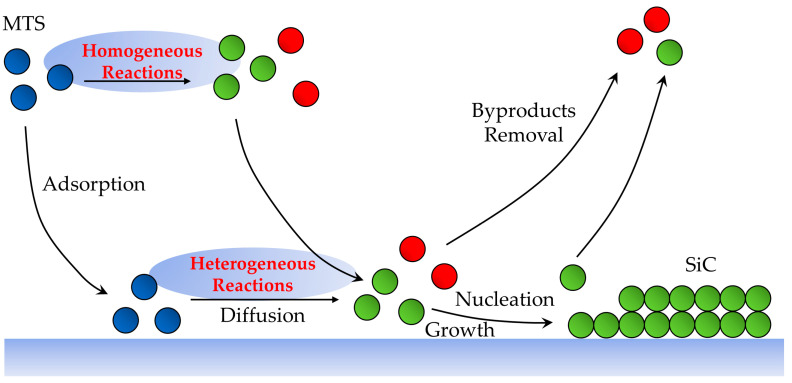
Schematic diagram of elementary steps during typical CVD process (taking SiC CVD process from MTS gas as an example): First, MTS molecules (bule spheres) are transported into the reactor. Then there are two possible reactions: homogeneous reaction and heterogeneous reaction. MTS molecules deposit directly or on the substrate, forming SiC molecules (green spheres) and byproducts (red spheres). SiC molecules undergo surface diffusion, nucleation, and growth before the formation of thin films. Finally, byproducts are removed.

To sum up, the elementary steps of the CVD process are shown in Figure 1.

## 3. Parameters in the CVD Process

The quality, uniformity, and characteristics of the deposited film heavily depend on various process parameters, such as temperature, pressure, precursors, reactions, substrate, and gas flow dynamics [12], as summarized in Table 1. Understanding and controlling these parameters is essential for optimizing the whole CVD process.

### 3.1. Temperature

Temperature is one of the most critical parameters in the CVD process. Reaction kinetics, precursor decomposition, and the properties of the deposited film are affected by temperature [45]. Wagner et al. [46] investigated the effects of deposition temperature on microstructure, crystal orientation, and texture of CVD TiN coatings, finding that the coatings yield different orientations and properties between 850 and 1050 °C. Kostogrud et al. [47] investigated the effects of temperature on the number of graphene layers and the defect rate of graphene planes during CVD graphene growth. Reep et al. [48] found three distinct temperature-dependent regions in CVD GaAs growth and proposed a model for the epitaxial growth of GaAs by the organometallic process. Johnson et al. [49] investigated the kinetics of CVD SiC growth, and surface decomposition mechanisms under different temperatures. Nakaso et al. [45] investigated the effects of reaction temperatures on the primary particle, morphology, and crystallinity during CVD preparation of TiO_2_. Fukushima et al. [50] investigated the influence of growth temperature on CVD SiC growth rate. Zeng et al. [51] investigated the effects of deposition temperatures on the phase composition, microstructure, and mechanical properties of TiB_x_N_y_ coatings during CVD preparation of TiB_x_N_y_. Hu et al. [52] investigated the effects of temperature on deposition rate, microstructure, and ablative properties of SiC coatings during CVD SiC growth.

### 3.2. Pressure

Pressure within the CVD chamber influences the mean free path of gas molecules, the rate of precursor delivery to the substrate, and the overall deposition rate. Wu et al. [53] investigated the effects of deposition pressure on the microstructure and mechanical properties of TiAlSiN coatings during CVD TiAlSiN preparation. Liang et al. [54] investigated the effects of deposition pressure on the diamond grain size and grain boundary fraction during CVD diamond film preparation. Ji et al. [55] investigated the effects of growth pressure on the void formation, filling rate, and filling efficiency of a 4H-SiC trench during the CVD process. Gakis et al. [56] investigated the effects of pressure on the catalyst activity and deposition uniformity during CVD preparation of CNTs. Bakr et al. [57] investigated the effects of deposition pressure on the structural, optical, and electrical properties of hydrogenated nanocrystalline SiC thin films during CVD preparation of SiC. Abbadie et al. [58] found that the variation in ZnO growth direction is triggered by furnace pressure.

### 3.3. Precursor Chemical/Physical Properties

The chemical/physical properties of precursor gases, including precursor volatility, reactivity, and purity, are fundamental to the CVD process, as they directly influence the reaction pathways, film composition, and deposition efficiency. The precursors need to be volatile and stable enough. Pasin et al. [59] investigated the effects of precursor volatility on delivery rate. Kada et al. [60] found that CpAllylNi is a better precursor than MeCp_2_Ni due to a higher volatility for producing Ni film. The chemical reactions on the substrate surface are greatly determined by the reactivity of the precursor. Highly reactive precursors may lead to higher deposition rates but can also increase the possibility of side reactions. Koponen et al. [61] investigated the precursors’ reactivities for the precursor design in the CVD process. Emslie et al. [62] investigated the reactions between metal and co-reactants to develop new pulsed-CVD reactivities. Impurities in the precursor can lead to unwanted elements in the film, thus affecting its properties. High-purity precursors are essential in developing high-quality films. Zhao et al. [63] investigated the effects of precursor purity on the quality of CVD h-BN films.

### 3.4. Gas Flow Dynamics

The flow dynamics of precursor and carrier gases affect the delivery of reactants to the substrate surface and the overall deposition process. Maintaining an appropriate flow rate is crucial for ensuring the deposition rate and film uniformity [64]. Ren et al. [65] found that a high flow rate of methane enhanced the deposition rate but reduced the grain size of the HfC-ZrC coating. Liu et al. [66] investigated that some issues such as low density, inhomogeneous thickness, and morphology of MoS_2_ can be overcome by adjusting the flow rate of carrier gas during CVD MoS_2_ growth. Kumar et al. [67] investigated the effects of C_2_H_2_ flow rates on the morphology, crystallinity, and mechanical properties of CVD SiCN thin films. Tripathi et al. [68] investigated the effects of C_2_H_2_ flow rate on the quality, diameter, and length of CNTs during CVD preparation of CNTs. Gakis et al. [56] investigated the effects of acetylene flow rate on the deposition uniformity of CNTs prepared by CVD processes. Das et al. [69] investigated the effect of N_2_ flow rate on the morphology, microstructure, electrochemical, and nanomechanical properties of TiCN films prepared by CVD.

Gas flow rate also influences the flow pattern structure, which has many effects on the CVD process. It has been reported that a laminar flow in the chamber is preferred for uniform deposition, as it ensures that reactants reach the substrate evenly. A turbulent flow can lead to uneven deposition and reduce film quality. Santen et al. [70] investigated the effects of turbulence on the CVD process in the chamber by numerical simulation methods. Kavousanakis et al. [71] investigated the effects of self-sustained periodic flows in the laminar flow regime on the film thickness during CVD process.

In some CVD processes, the deposition rate is limited by the diffusion of precursor gases to the substrate. The mass transport can be enhanced and the deposition efficiency can be improved by optimizing gas flow and pressure. The thickness of the flow boundary layer affects the transport of precursors to the surface. Reducing the flow boundary layer thickness through optimized flow conditions can enhance deposition rates. Croon et al. [72] derived deposition rate equations especially when the surface reaction rates were limited under the operating conditions of flow boundary layer. Singh et al. [73] investigated the role of concentration boundary layer on large-area CVD MoS_2_ growth.

### 3.5. Substrate Surface Properties

The condition of the substrate surface plays a significant role in determining the adhesion, morphology, and overall quality of the deposited film, considering its effects on nucleation, surface diffusion, and film orientation [74].

The substrate must be thoroughly cleaned to remove organic residues, oxides, and other contaminants that could interfere with film growth or lead to defects [75,76]. In some cases, the substrate may require chemical or plasma treatments to enhance adhesion and promote uniform nucleation. Saito et al. [77] proposed a new method using an in situ silane cleaning treatment on native oxide reduction to increase titanium silicide nuclei and silicon consumption control. Yin et al. [78] introduced the pre-treatments of SiO_2_/Si, Si, and quartz glass substrates for CVD preparation of MoS_2_ and WS_2_. Yu et al. [79] investigated the effects of substrate pre-treatment on the CVD synthesis of diamond films. Jeon et al. [33] introduced a SiO_2_ substrate with the oxygen plasma process for CVD growth of large-area 2D MoS_2_ films.

Surface roughness can influence nucleation density and growth mode of the film. A smoother surface generally promotes uniform nucleation and growth, while a rough surface may lead to non-uniformities, thus affecting the optical, electrical, and mechanical properties of the thin films. Mallik et al. [80] investigated the effects of substrate roughness on the nucleation and growth of diamond films prepared by PECVD. Luo et al. [81] investigated the effects of substrate roughness and feedstock concentration on CVD growth of large-area graphene. Mallik et al. [82] investigated the substrate roughness on the growth of polycrystalline diamond films prepared by HFCVD.

The surface energy of the substrate affects the adhesion of the deposited film. Surface energy also influences the distribution and density of nucleation sites, influencing the microstructure and grain size of the film. Khan et al. [83] introduced a low-temperature PECVD technique to enhance the direct CVD growth of graphene on dielectric substrates due to their low surface energy. Godin et al. [84] increased the polar surface energy of SiO_2_ substrate by oxygen plasma treatment to increase the average grain size and surface coverage for CVD growth of monolayer WS_2_.

**Table 1:** Typical parameters that influence the properties of the deposited films.

Parameter	Object	Form	Conclusions	Reference
Temperature	TiN	Nitride ceramic coating	850 °C: fine-grained lenticular-like structures; 900 °C: pyramidal morphology; 950 °C: (110) oriented films of facetted structure; 1000 °C: (100) oriented films of facetted structure; 1050 °C: irregular facetted crystal without texture.	Wanger et al. [46]
	Graphene	Carbon material coating	With an increase in temperature, the number of layers of graphene coatings decreases, the defectiveness and the degree of copper coverage by graphene increases.	Kostogrud et al. [47]
	GaAs	Semiconductor coating	Low-temperature: kinetic control; Mid-temperature: mass transport limited; High-temperature desorption limited.	Reep et al. [48]
	TiO_2_	Metal oxide ceramics nanoparticle	The reaction temperature at which the smallest primary particle diameter occurred matched the temperature where the precursor fully converted.	Nakaso et al. [45]
	TiBN	Nitride boride composite ceramics coating	830 and 860 °C: particle-like surface; 890 and 920 °C: needle-like surface; With the increase in deposition temperature, the hardness of the coating decreases.	Zeng et al. [51]
	SiC	Semiconductor coating	900–1000 °C: smooth and spherical compact packing morphology; 1100–1200 °C: irregular cauliflower morphology. 1000 °C: suitable density, roughness, and uniform size of coating microstructure.	Hu et al. [52]
Pressure	TiAlSiN	Nitride ceramic coating	With increasing pressure, the coating changed from columnar to featureless grains.	Wu et al. [53]
	Diamond	Carbon material coating	With decreasing growth pressure, diamond grain sizes reduce, surface roughness reduces, and the coating morphologies changes from columnar to grainy structures. The pressure has prominent influence on the film growth rate.	Liang et al. [54]
	4H-SiC	Super junction	Increasing the growth pressure from 10 to 38 kPa, growth around the mesa surface reduces, which lessens the risk of void formation; and a high filling rate as well as an improved filling efficiency, which is up to 7.	Ji et al. [55]
	CNT	Nanotube	At higher pressure, the uniformity of deposited CNTs decreases along the wafer, with the effect being more significant at elevated temperature.	Gakis et al. [56]
	ZnO	Nanostructures	The pressure influences the size, the shape of the structures, and the formation probability of the nuclei.	Abbadie et al. [58]
Precursor properties	Ni	Metal coating	CpAllylNi is a better precursor than MeCp_2_Ni due to a higher volatility.	Kada et al. [60]
	/	/	The reactivity of the precursors must exhibit a careful balance of non-reactivity in the gas phase and selective reactivity at the surface.	Koponen et al. [61]
	/	/	Provide a unique reactivity-based perspective of metal ALD/pulsed-CVD.	Emslie et al. [62]
	h-BN	Ceramic coating	High-purity precursor is essential for the reproducible growth of the large area, smooth, and continuous h-BN layers.	Zhao et al. [63]
Gas flow dynamics	HfC-ZrC	Composite carbide ceramics coating	A high flow rate of methane enhances the deposition rate but reduces the grain size of the HfC-ZrC coating.	Ren et al. [65]
	MoS_2_	Transition-metal dichalcogenide (TMD) coating	The variation of carrier gas flow rate could overcome the low density, the inhomogeneous thickness, and morphology of MoS_2_.	Liu et al. [66]
	SiCN	Composite ceramics coating	As C_2_H_2_ flow rate increases, the carbon content in SiCN rises, the film’s corrosion resistance enhances, and the mechanical properties of the film improve.	Kumar et al. [67]
	CNTs	Nanotube	The yield, diameter, and length of CNTs increase with flow rate of C_2_H_2_ up to 20 sccm. Beyond 20 sccm, yield and length start decreasing but increasing in diameter.	Tripathi et al. [68]
	TiCN	Composite ceramics coating	With an increase in N_2_ flow rate, the roughness of the coating increases, the lattice parameter of the coating decreases, the corrosion resistance of the coating improves, and the hardness of the coating increases.	Das et al. [69]
Substrate surface properties	TiSi_2_	Metal silicide coating	An in situ silane cleaning treatment greatly increases titanium silicide nuclei, and a silicon consumption control.	Saito et al. [77]
	MoS_2_ and WS_2_	TMD coating	Due to the lattice matching between 2D materials and substrate, defect/impurities on the interface, and conductance/transparency of different substrates, the morphology, crystalline quality, and optical properties of the coating will change.	Yin et al. [78]
	Graphene	Carbon material coating	Surface morphology of the catalytic Cu substrate and the concentration of carbon feedstock gas are crucial in the homogeneity and electronic transport properties of the final graphene film.	Luo et al. [81]
	MoS_2_	TMD coating	Layer-controlled and large-area CVD MoS_2_ films can be achieved by treating the surfaces of their bottom SiO_2_ substrates with the oxygen plasma process.	Jeon et al. [33]
	Diamond	Carbon material coating	The roughness created by the argon plasma treatment of the Si substrate surfaces enhances the nucleation and growth behaviors of the diamond films.	Mallik et al. [82]
	Graphene	Carbon material coating	To achieve controlled graphene growth on dielectric and semiconducting substrates, it is crucial to thoroughly understand the metal-catalyst-free direct CVD growth mechanism of graphene on these surfaces.	Khan et al. [83]
	WS_2_	TMD coating	Oxygen plasma treatment of the substrate increases the average domain size of WS_2_ monolayers, but nucleation density reduces slightly, and monolayer surface coverage increases.	Godin et al. [84]

## 4. CVD Models in Different Scales

CVD is a complex process, and it is challenging to achieve controlled and reproducible growth on a large scale. Great efforts have been made to establish comprehensive mathematical models. In this section, several numerical models in different scales will be introduced in detail.

### 4.1. Nanoscale

Nanoscale models, including density functional theory (DFT) and molecular dynamics (MD), play a crucial role in CVD simulations by providing detailed insights into precursor chemistry, surface adsorption and reactions, nucleation, and growth.

#### 4.1.1. Density Functional Theory

**Figure 2:**
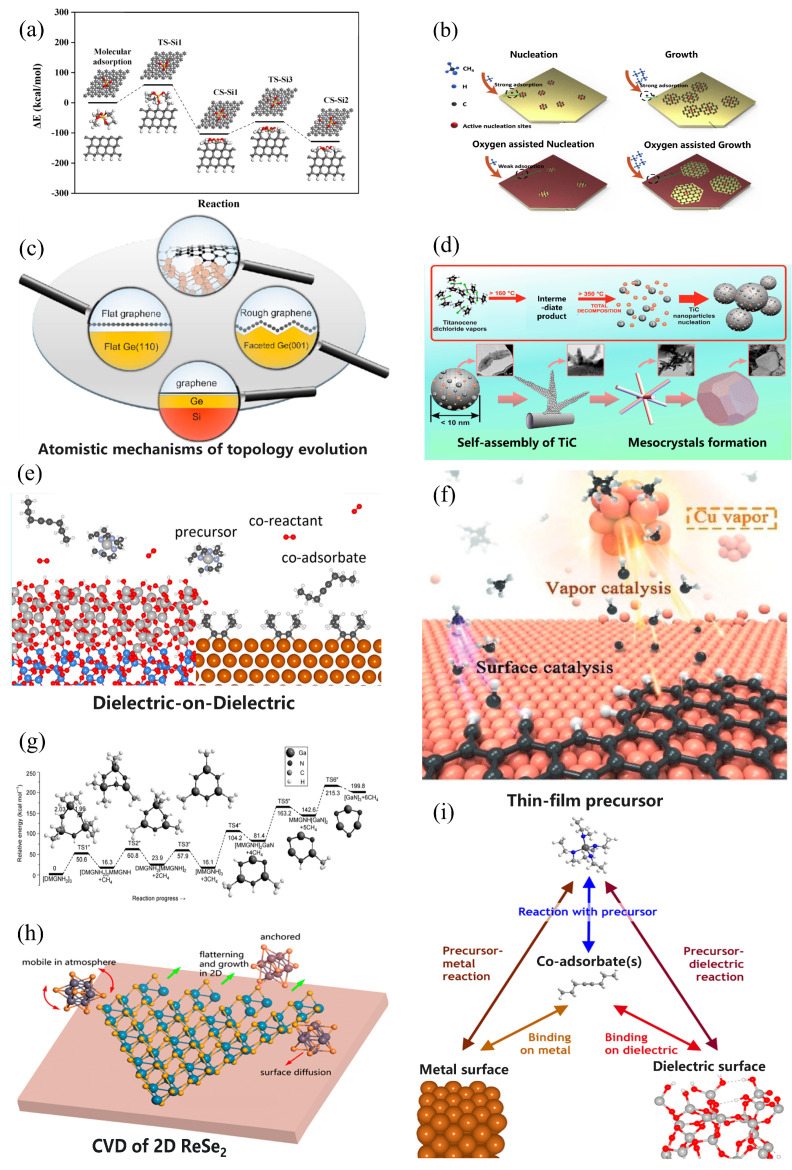
Schematic diagrams of typical CVD simulations using DFT: (**a**) continued reaction process for a tetraethyl orthosilicate molecule to dissociate and chemisorb on the surface of diamond (1 1 1), adapted from [95]; (**b**) effects of O_2_ adsorption during nucleation and growth on the synthesis of graphene, adapted from [97]; (**c**) CVD growth of large-scale graphene and the atomistic mechanisms of graphene evolution in different Ge substrates, adapted from [116]; (**d**) Cp_2_TiCl_2_ pyrolysis, self-assembly paths of TiC mesocrystal, and the effects of the organization of nanoparticles, adapted from [90]; (**e**) the use of 4-octyne as a co-adsorbate in CVD preparation of ZrO_2_ thin films on SiO_2_ and Cu substrates combined with DFT calculations, adapted from [92]; (**f**) Cu vapor and its components evaporated from the substrate and two typical catalytic processes involved in CVD graphene growth process, adapted from [98]; (**g**) potential energy surfaces of the [DMGNH_2_]_3_ decomposition path calculated by DFT method, adapted from [91]; (**h**) the debated CVD growth of 2D ReSe_2_, and the existence of stable intermediate species, adapted from [100]; (**i**) the interactions must be considered focusing on thin-film precursor and the co-adsorbate species, adapted from [92].

Density functional theory (DFT) is a computational quantum mechanical model investigating the electronic structure of many-body systems. The gas reactions, surface adsorption, surface chemical reactions, and nucleation/growth in the CVD process can be simulated by DFT [85], as summarized in Figure 2.

##### Precursor Gas Reactions

The thermodynamic parameters such as enthalpy, entropy, and Gibbs energy as well as kinetic parameters like reaction rate constant can be calculated by DFT to study precursor gas reactions in the CVD process. Timoshkin et al. [86] investigated the dissociation and elimination of various gas phase precursors during CVD preparation of GaN. Won et al. [87,88,89] investigated the gas phase decomposition mechanism of the precursor during CVD preparation of TaN, W_2_N, and ZrC. Vilkov et al. [90] studied the decomposition mechanisms of the gas-phase reactions of the precursor during CVD preparation of TiC. Shi et al. [91] investigated the reaction mechanism of precursor adducts during CVD preparation of GaN. The results show that DFT simulations can analyze the thermal stability of CVD precursors and their reaction intermediates and products, and also help to predict the reaction paths of the precursor gas reactions.

##### Surface Adsorption

The surface adsorption in the CVD process can be investigated by calculating the energy parameters such as adsorption energy, desorption energy, and bonding energy by DFT simulations. Suh et al. [92] investigated the regional selective adsorption mechanism of 4-octyne on the surface of Cu and SiO_2_ during CVD preparation of ZrO_2_. Saedy et al. [93] investigated the preferential adsorption mechanism of Co on Pt surface during CVD preparation of Pt_3_Co. Karlsson et al. [94] investigated the adsorption mechanism of BF_x_ and NH_x_ on the surface of BN during CVD preparation of BN. Lu et al. [95] investigated the adsorption mechanism of acetone and methane on diamond surface during CVD preparation of diamond. Popov et al. [96] investigated the adsorption mechanism of C atoms on Cu surface during CVD preparation of graphene. Chen et al. [97] investigated the mechanism by which oxygen promoted the adsorption of C atoms on Cu surface during CVD preparation of graphene. The results show that DFT simulations can analyze the adsorption mechanism of various precursors and intermediates in CVD process.

##### Surface Chemical Reactions

Surface chemical reactions usually occur on gas-phase precursors or solid surfaces such as substrates and catalysts. Energy parameters such as reaction energy, adsorption energy, and activation energy can be calculated by DFT simulations and energy diagrams or potential energy diagrams can be drawn to study the surface chemical reactions in the CVD process.

Sun et al. [98] investigated the catalytic mechanism of the precursor in CVD preparation of graphene on the surface of Cu clusters. Ran et al. [99] investigated the chemical behaviors of water molecules on the surface of Ni catalysts during CVD preparation of carbon nanotubes. Chen et al. [100] investigated the key reaction steps of the precursors on ReSe_2_ surface during CVD preparation of ReSe_2_. Wang et al. [101] investigated the decomposition mechanism of NH_3_ on Si surface during CVD preparation of Si_3_N_4_. Nakajima et al. [102] investigated the reaction mechanism of the precursors on Al surface during CVD preparation of Al. Filatova et al. [103] investigated the reaction mechanism of various precursors with bare and H-terminated 3C-SiC (011) during CVD preparation of SiC. Lu et al. [95] investigated the decomposition mechanism of acetone and methane on diamond surface during CVD preparation of diamond. Tanaka et al. [104] investigated the reaction mechanism of TiCl_4_ and NH_3_ on SiO_2_ surface during CVD preparation of TiN. The results show that DFT simulations are very helpful in predicting the reaction paths of surface chemical reactions.

##### Nucleation and Growth

Energy parameters such as reaction energy, diffusion energy, nuclear energy, and activation energy can be calculated by DFT simulations, and energy diagrams or potential energy diagrams can be drawn. Charge density, local state density, Bader charge, and other charge parameters can also be calculated to study the nucleation and growth in CVD process.

Li et al. [105] investigated the influences of different surfaces (Fe, Co, Ni, Cu) on the diffusion and nucleation of C atoms during CVD preparation of carbon nanotubes and graphene. Keyshar et al. [106] investigated the origin of dendritic shapes on ReS_2_ surface during CVD preparation of ReS_2_. Kovalenko et al. [107] investigated the mechanism of Ni center formation of graphene on Ni surface during CVD preparation of Ni-doped graphene. Zhang et al. [108] investigated the effects of different hydrocarbons on the formation mechanism of carbon nanotubes during CVD preparation. Dabrowski et al. [109] investigated the initial states of graphene growth on Ge surfaces during CVD preparation of graphene. Yu et al. [110] investigated the effects of Se introduction on MoS_2_ nucleation and growth during CVD preparation of MoS_2(1-x)_Se_2x_ alloy. Lawson et al. [111] investigated the influence of different metal oxide surfaces (Al_2_O_3_, HfO_2_, MgO) on the nucleation of MoF_6_ during CVD preparation of MoF_6_. Kimura et al. [112] investigated the effects of oxygen-containing additives on the etching mechanism of carbon nanotube growth during CVD preparation. Tsakonas et al. [113] investigated the rate-determining steps of the graphene growth process during CVD preparation of graphene. Zhu et al. [114] investigated the nucleation mechanism of BN on different transition metal surfaces (Ag, Cu, Pt, Co) during CVD preparation of BN. Jian et al. [115] investigated the growth mechanism of vapor-dissociated solids (VDS) of graphite on ZnO surface during CVD preparation of graphite. The results show that DFT simulations can not only study nucleation and growth processes from the perspective of energy, but also help to understand the mechanism of nucleation and growth processes from an electronic scale.

#### 4.1.2. Molecular Dynamics

**Figure 3:**
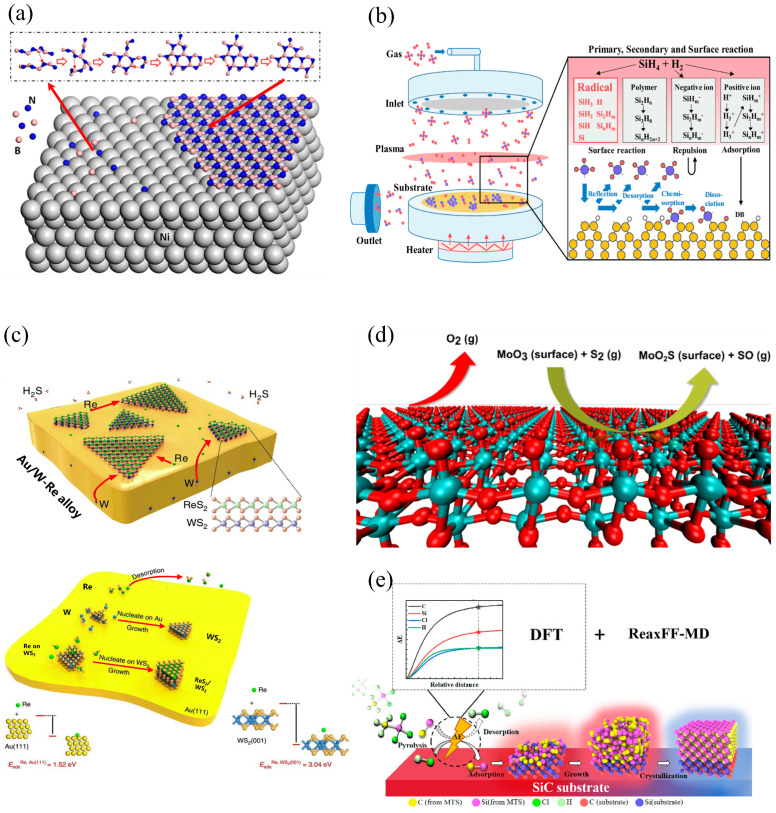
Schematic diagrams of typical MD CVD simulations: (**a**) elementary nucleation and growth processes of a hBN monolayer structure from elemental B and N, adapted from [118]; (**b**) deposition processes of Si thin films by PECVD technique using SiH_4_ and H_2_ as source gases, adapted from [132]; (**c**) mechanism and modeling of the growth of an out-of-plane 2D ReS_2_/WS_2_ heterostructure, adapted from [139]; (**d**) reaction mechanisms and kinetics of the sulfidation of MoO_3_ surfaces using S_2_ gas precursors, adapted from [133]; (**e**) detailed analysis of CVD process of preparing SiC materials using ReaxFF-MD, adapted from [140].

Molecular dynamics (MD) is a classical mechanics-based method used to analyze the motions of atoms or molecules in a system over time. Newton’s equations are numerically solved to determine the trajectories of atoms or molecules. Interatomic potentials or force fields are used to calculate the inter-particle forces and potential energies. MD can provide an insight into nucleation and growth, surface chemical reactions, and surface adsorptions [85], as shown in Figure 3.

##### Nucleation and Growth

MD can demonstrate the nucleation and growth during CVD process at microscopic level. Nucleation and growth can be investigated through surface structure evolutions (such as atomic rings, atomic chains, atomic clusters) and energies (such as binding energy, formation energy, adsorption energy, and diffusion energy).

Meng et al. [117] investigated the evolution of carbon structure at different temperatures during CVD preparation of graphene and the growth dynamics of graphene on Ni surface. Liu et al. [118] investigated the nucleation and growth mechanism of BN on Ni surface during CVD preparation of BN. Wu et al. [119] investigated the effects of C/H ratio in hydrocarbon precursors on graphene CVD growth. Chen et al. [120] investigated the dissolution and precipitation mechanism of graphene on Ni surface at the initial growth stage during CVD preparation of graphene, as well as the dynamic evolutions of graphene nucleation. Zhang et al. [121] investigated the growth mechanism of graphene on Cu surface during CVD preparation of graphene. Shariat et al. [122] investigated the promoting effects of low-temperature plasma on carbon nanotubes CVD growth. Xu et al. [123] investigated the growth stability of graphene nanostructures on semi-molten Cu substrate during CVD preparation of graphene. Shibuta et al. [124] investigated the graphitization ability of different transitional metal substrates during CVD preparation of carbon nanotubes. Eveleens et al. [125] investigated the influence of ammonia on the nucleation of carbon nanotubes on iron catalyst during CVD preparation of carbon nanotubes. Lu et al. [126] investigated the graphene formation and growth mechanism on Ni surfaces by using the polycyclic aromatic hydrocarbon carbon sources during CVD preparation of graphene, and investigated the effects of annealing temperature, precursor concentration, and surface type. McLean et al. [127] investigated the nucleation mechanism of BN nanotubes formed on Ni surface by ammonia borane precursors during CVD preparation of BN nanotubes. Rasuli et al. [128] investigated the effects of substrate temperatures, C flow rates, and C flow energies on the growth of graphene on Ni surface during CVD preparation of graphene. Paul et al. [129] investigated the effects of the substrate’s crystallinity on the growth orientation of MoS_2_ prepared by CVD. Momeni et al. [130] investigated the effects of binding energy and equilibrium distance of WSe_2_ and different substrates on the quality and uniformity of WSe_2_ monolayer films.

##### Surface Chemical Reactions

Surface chemical reactions during the CVD process can be investigated by calculating the reaction rate, bonding/breaking evolution, reaction product evolution, and reaction energy. Zhang et al. [131] investigated the catalytic dehydrogenation reactions of the precursor C_2_H_2_ on SiC surface during CVD preparation of graphene. Uene et al. [132] investigated the effects of Si substrate temperature and H coverage on the surface reactions of silane during CVD preparation of Si. Hong et al. [133,134,135] investigated the sulfurization mechanism of MoO_3_ by S_2_ precursors and MoO_3_ by pure H_2_S and H_2_S/H_2_ mixture precursors during CVD preparation of MoS_2_. Mishra et al. [136] investigated the sulfurization mechanism of MoO_3_ by H_2_S precursors during CVD preparation of MoS_2_.

##### Surface Adsorption

The surface adsorption in the CVD process can be investigated by calculating the adsorption rate, adsorption coefficient, adsorption/desorption type, and its probability by MD. Ito et al. [137] investigated the effect of C/H ratio on adsorption rate in hydrocarbon precursors during CVD preparation of amorphous carbon. Schwaederlé et al. [138] investigated the influences of substrate temperature and surface hydrogenation on the adsorption coefficient of precursor CH_3_ on diamond surface during CVD preparation of diamond.

### 4.2. Mesoscale

Mesoscale refers to the size between nanoscale and macroscale [141]. Nanoscale models have excessive nanoscopic freedom degrees, requiring extremely small time-steps to resolve, making it unfeasible for long-time simulations. Therefore, mesoscale models are required, including phase-field model and kinetic Monte Carlo model.

#### 4.2.1. Phase-Field Model

**Figure 4:**
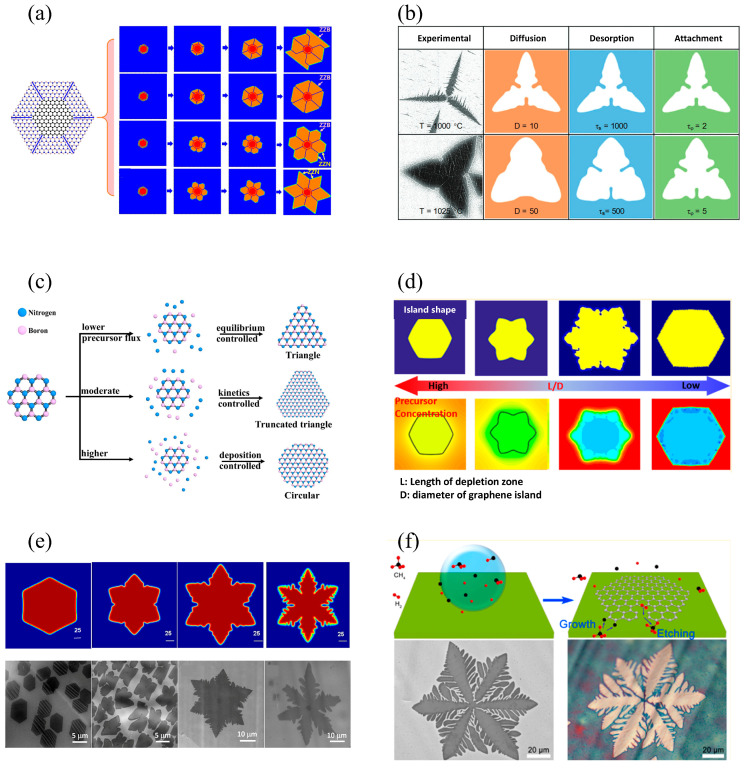
Schematic diagrams of typical phase-field CVD simulations: (**a**) growth process of G-h-BN flakes with an interface between the graphene and h-BN comprising C-N bonds along ZZ directions, adapted from [142]; (**b**) phase-field simulation in comparison with experimental results during CVD hBN growth, adapted from [144]; (**c**) the hypothesis of shape evolutions of hBN under different precursor flux, adapted from [146]; (**d**) morphology evolutions of graphene during CVD growth under the influence of carbon precursors, carbon flux, and precursor concentration on the metal surface, adapted from [147]; (**e**) changes in the morphology of graphene islands from simulations with increasing O concentration, adapted from [151]; (**f**) etching-controlled growth and synthesis of graphene with various morphologies calculated by PF simulations, adapted from [157].

Phase-field (PF) models are mainly used to investigate the nucleation and growth in the CVD process. It can be used to study the nucleation and growth processes by calculating morphological evolution (typical snowflake diagram), concentration field evolution, density field evolution, and polar diagrams, as shown in Figure 4. Geng et al. [142] investigated the growth mechanism of graphene-BN on Cu substrate during CVD preparation of graphene-BN heterostructures. Naylor et al. [143] investigated the growth mechanism of 1T’ morphology with spike-shaped islands during CVD preparation of 1H-2 and 1T’-2 heterostructure. Li et al. [144] investigated the growth mechanism of BN on Cu-Si alloy surface during CVD preparation of BN. Huang et al. [145] investigated the dendrite growth mechanism of TiO_2_ during CVD preparation. Liu et al. [146] investigated the nucleation and growth mechanism of BN on Cu substrate during CVD preparation of BN. Zhuang et al. [147] investigated the growth mechanism of various graphene islands during CVD preparation of graphene. Qiang et al. [148] investigated the nucleation and growth mechanism of WS_2_ on SiO_2_/Si substrate during CVD preparation of WS_2_. Chowdhury et al. [149] investigated the role of H in suppressing secondary nucleation of MoS_2_ on SiO_2_/Si surface during CVD preparation of MoS_2_. Meca et al. [150] investigated the effects of H_2_ on CVD graphene growth. Srinivasan et al. [151] investigated the oxygen-promoted effects on CVD graphene growth on Cu substrate. Fashu et al. [35] investigated the growth mechanism of CVD graphene growth. Roy et al. [152] investigated the nucleation and growth mechanism of MoSe_2_ on SiO_2_/Si surface during CVD preparation of MoSe_2_. Li et al. [153] investigated the grain boundary growth mechanism during CVD preparation of polycrystalline graphite. Xu et al. [154] investigated the influences of adsorption atom concentration distributions on CVD MoS_2_ growth. Elder et al. [155] investigated the effects of H on CVD graphene growth. Meca et al. [156] investigated the epitaxial graphene growth and shape dynamics on Cu substrate. Luo et al. [157] investigated the etching effects on CVD graphene growth. Li et al. [158] investigated the differences in growth mechanism between bilayer and monolayer graphene during CVD preparation of graphene. Wu et al. [159] investigated the spiral growth mechanism of CVD preparation of SnSe_2_.

#### 4.2.2. Kinetic Monte Carlo

**Figure 5:**
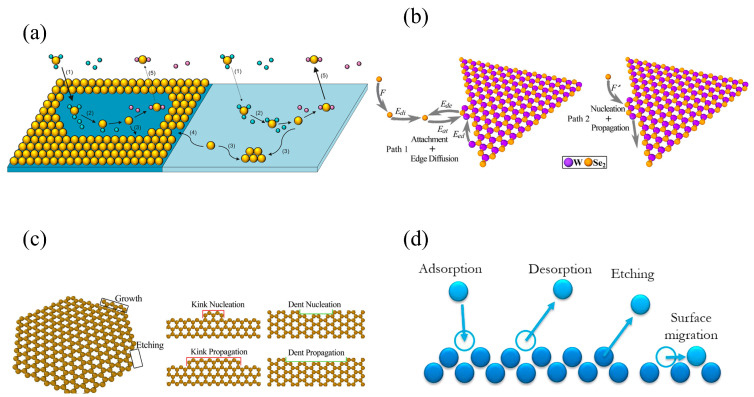
Schematic diagrams of typical KMC CVD simulations: (**a**) elementary processes involved in Ru CVD, and the surface diffusion process calculated by KMC method, adapted from [163]; (**b**) two possible paths for the ultrafast growth of triangular WSe_2_ domains, adapted from [164]; (**c**) KMC model and two typical nucleation and propagation processes for the growth and etching of graphene during CVD, adapted from [185]; (**d**) processes considered in 3D KMC models during the growth of diamond, adapted from [184].

Kinetic Monte Carlo (KMC) method is used to predict the kinetic mechanisms and growth morphology of 2D materials. It creates an event catalog including all possible kinetic events, which are selected randomly depending on their activation energies [160] as summarized in Figure 5.

Nucleation and growth processes can be investigated by using KMC simulations to calculate surface structure evolution, deposition rate, growth rate, diffusion rate, surface roughness, and density. Tsalikis et al. [161] investigated the influence of gas-phase precursor components on CVD Si growth. May et al. [162] investigated the effects of adsorption, desorption, and surface diffusion on CVD diamond growth rate. Grillo et al. [163] investigated the effects of surface diffusion on selective deposition during CVD preparation of Ru. Chen et al. [164] investigated the nucleation and growth mechanism of CVD WSe_2_ preparation. Liu et al. [165] investigated the influences of different SiC surfaces and temperatures on CVD SiC growth. Akter et al. [166] investigated the growth mechanism of porous SiO_2_ on non-porous SiO_2_ surface during CVD preparation of porous SiO_2_. Battaile et al. [167] investigated the influences of different diamond surfaces and temperatures on CVD diamond growth. Jiang et al. [168] investigated the growth mechanism of graphene on an active catalyst surface with graphene/substrate lattice mismatch during CVD preparation of graphene. Grujicic et al. [169] investigated CVD carbon nanotubes growth mechanisms. Förster et al. [170] investigated the growth rates of carbon nanotubes of different chiralities during CVD preparation of carbon nanotubes. Balbuena et al. [171] investigated the effects of temperature on CVD Si growth. Sun et al. [172] investigated the growth and formation mechanism of adlayer graphene spirals during CVD preparation of graphene. Bouhadiche et al. [173] investigated the influences of precursor composition and temperature on the size and density of Si clusters during CVD preparation of SiN_x_. An et al. [174] investigated the influence of Cl atoms on CVD diamond growth. Göltl et al. [175] investigated the growth mechanism on Ge surface during CVD preparation of graphene. Liu et al. [176] investigated the effects of different SiC surfaces on CVD growth rate of SiC. Zhu et al. [177] investigated the mechanism of CVD GaInP growth. Fan et al. [178] investigated the influences of different Cu substrates on CVD graphene growth. Budagosky et al. [179] investigated the growth mechanism of amorphous TiO_2_ and polycrystalline ZnO. Zhang et al. [180] investigated the influences of precursor composition and temperature on deposition rate during CVD preparation of diamond. Wu et al. [181] investigated the anisotropic growth mechanism of WS_2_ on SiO_2_ surface during the CVD preparation of WS_2_. Liu et al. [182] investigated the effect of temperature on the growth rate during CVD preparation of SiC. Liu et al. [183] investigated the effect of MTS concentration on CVD SiC growth rate. Valentin et al. [184] investigated the step-flow growth mechanism of CVD diamond preparation.

### 4.3. Macroscale

**Figure 6:**
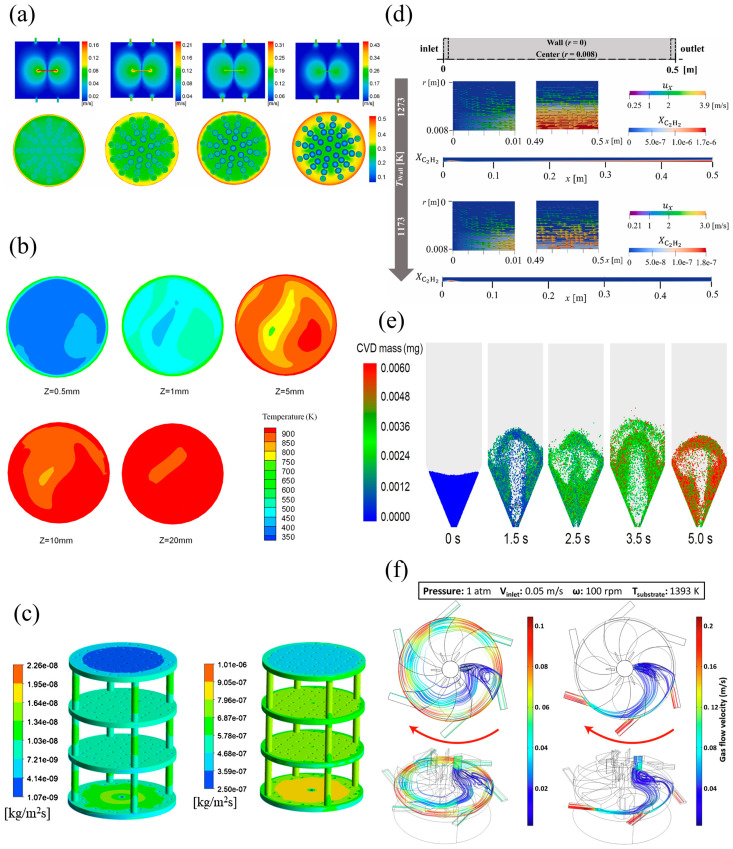
Schematic diagrams of typical CVD simulations using CFD method: (**a**) magnitude of the gas flow velocities and SiC deposition mass fractions on the graphite plate under different gas flow rates, adapted from [187]; (**b**) simulated temperature distributions near the inlet region during CVD preparation of Si, adapted from [191]; (**c**) C_2_H_2_ mole fractions at different cross-sections of the reactor and deposition rates on the substrate, adapted from [195]; (**d**) C_2_H_2_ mole fractions with velocity vectors near the inlet and exit regions and C_2_H_2_ mole fraction inside the reactor at 1173 K and 1273 K, adapted from [197]; (**e**) particle coating process simulated by CFD–DEM–CVD model in a spouted bed during the preparation of TRISO particles, adapted from [199]; (**f**) velocity streamlines obtained by CFD method to demonstrate effect of gas type on flow in CVD reactor chamber design, adapted from [202].

Macroscale models play a crucial role in the simulation and optimization of the CVD process, providing insights into the behaviors of the system on a reactor scale. These models focus on the parameters like gas flow, heat transfer, mass transport, and chemical reactions. By capturing the global behaviors of the CVD process, macroscale models help in designing reactors, optimizing operating conditions, and predicting the overall performances of the deposition process. Computational fluid dynamics (CFD) models are commonly used in the simulations of the CVD process. CFD models provide detailed information about the velocity, pressure, temperature, and concentration fields during the deposition process, as shown in Figure 6.

#### 4.3.1. Gas Flow Dynamics

Gas flow is influenced by chamber geometry and thermal gradients between the inlet and the reactor [186]. The distributions of precursor gases, carrier gases, and byproducts are crucial for ensuring uniform depositions on the substrate. CFD is used to calculate the gas flow dynamics in the reactor. Cao et al. [187] investigated the gas-flow field distributions inside the reactor by using the CFD method during large-size graphite plate/SiC preparation by CVD. Li et al. [188] investigated the distribution of gas flow rate near the substrate in CVD reactor by using the CFD method. Yu et al. [189] investigated the flow distribution inside the CVD reactor by using the CFD method.

#### 4.3.2. Heat Transfer

Heat transfer is another critical aspect of the CVD process, as temperature directly influences the reaction kinetics, precursor decomposition, and the quality of the deposited film. Liu et al. [190] investigated the temperature profile in high-speed deposition processes by CFD simulations during SiC fiber synthesis by CVD. Liu et al. [191] investigated the heat transfer and temperature distributions during simulated Si CVD by using the CFD–population balance model (PBM) method. Endo et al. [192] investigated the temperature distributions in a CVD reactor in order to establish favorable conditions for higher quality nanotubes.

#### 4.3.3. Mass Transport

Mass transport involves the movement of precursor gases and reactive species in the reactor. It is critical for determining the growth rate and uniformity of the deposited film. Vilá et al. [193] investigated the effects of precursor concentration gradient on the growth morphology of 2D MoS_2_. Zhang et al. [194] investigated the effects of the concentration gradient of Mo precursor on the morphology of MOS_2_ prepared by CVD. Gupta et al. [195] investigated the mole fraction of C_2_H_2_ species in CH_4_ pyrolysis process in a CVD reactor by using the CFD method.

#### 4.3.4. Chemical Reactions

CFD can be coupled with chemical reaction models to simulate the kinetics of the deposition process, allowing for a more accurate prediction of chemical reactions. He et al. [196] investigated the increase in reaction kinetics by the porous mediator using the CFD method. Ogawa et al. [197] proposed a SiC CVD growth model of a MTS/H_2_ system and investigated the pyrolysis reactions and SiC growth on the substrate. Liu et al. [198] investigated the silane pyrolysis kinetic during Si growth in fluidized bed (FB)–CVD by using the CFD–PBM method. In our research group, a CVD coating model is proposed to simulate the fluidized bed-chemical vapor deposition process, based on reaction product profiles calculated from chemical reactions [199].

In some CVD processes involving nanoparticle synthesis, it is important to consider particle behaviors like particle mixing or transport in the chemical reaction process. Liu et al. [200] predicted the evolution of TiO_2_ nanoparticles by using the CFD–PBM method. Liu et al. [201] investigated the particle coating process of tri-structural-isotropic (TRISO) particles prepared by FB–CVD using CFD–discrete element model (DEM)–PBM method.

#### 4.3.5. Reactor Design

CFD is an essential tool in the design and scale-up of CVD reactors, and can be used to predict reactor size, geometry, and operating conditions. Yousefian et al. [202] investigated the feasibility of a new vertical-type high-pressure CVD reactor designed for high-quality growth of group II nitrides that are high in content. Bijjargi et al. [203] designed a fuel combustion nozzle in the hot-wall CVD reactor for the deposition of SiC. Papavasileiou et al. [204] proposed an efficient CFD model for the design of an industrial CVD reactor.

## 5. Typical Multiscale Coupling CVD Models

Multiscale CVD models aim to simulate and understand the CVD process by integrating different spatial and temporal scales, allowing for a comprehensive analysis of the system’s behavior from nanoscopic to macroscopic levels. There are many works in the literature that have focused on this multiscale coupling topic, such as the following detailed below.

### 5.1. Nano-Meso Scale

In terms of nano-meso scale coupling, a typical case is DFT–KMC coupling. The energy parameters calculated by the DFT method can be used in KMC simulations to investigate the nucleation and growth during the CVD process. Chen et al. [205] investigated the graphene CVD preparation process by using the energy parameters calculated by the DFT method in KMC simulations. Gaillard et al. [206] investigated the graphene CVD preparation by using the diffusion energy calculated by the DFT method in KMC simulations. An et al. [207] investigated the CVD preparation process of AlN by using the activation energy of chemical reactions calculated by DFT in KMC simulations. Nie et al. [208] investigated the CVD preparation process of WSe_2_ by using the diffusion energy and adsorption energy calculated by the DFT method in KMC simulations.

### 5.2. Nano-Macro Scale

In terms of nano-macro scale coupling, DFT–CFD coupling, MD–CFD coupling, and DFT–MD–CFD coupling are typical cases. In DFT–CFD coupling, Liu et al. [200] investigated the evolution of TiO_2_ nanoparticles in the CVD process by using the DFT method to obtain the thermodynamic properties of the materials and the thermodynamics and kinetics of related chemical reactions, and then introduced the simplified descriptions of the relevant thermodynamic and kinetic data into the CFD–PBM model. The temperature field, velocity field, and particle size distribution in the CVD process were obtained. Zahi et al. [209] investigated Si CVD process by using the adhesion coefficient of the precursor on the substrate surface calculated by the DFT method in CFD simulations to obtain the density and radius of Si nanocrystals.

In MD–CFD coupling, Wang et al. [210] investigated the SiC CVD process. First, key deposition parameters such as gas flow rate, deposition pressure, and gas composition were calculated by MD method. Then, the results of MD simulations were applied to the CFD method to obtain the macroscopic flow field and deposition rate distribution.

In DFT–MD–CFD coupling, Xuan et al. [211] investigated the CVD preparation process of WSe_2_. Firstly, the parameters of the reaction force field were calculated by the DFT method and used in MD simulations. The possible reaction paths and kinetic parameters were then calculated by the MD method, and used in CFD simulations to investigate the main gas-phase processes and the growth behaviors of the film.

### 5.3. Meso-Macro Scale

In terms of meso-macro scale coupling, the typical cases are PF–CFD coupling and KMC–CFD coupling. In PF–CFD coupling, Ji et al. [212] investigated the BN CVD process by using the precursor concentration distributions calculated by the CFD method in PF simulations to calculate the distribution and morphology of BN. Momeni et al. [213] investigated the CVD preparation process of MoS_2_ and WSe_2_, and applied the precursor concentration calculated by the CFD method in PF simulations to obtain the morphological evolution of MoS_2_ and WSe_2_ on the substrate. Momeni et al. [214] investigated the CVD preparation of WSe_2_ monolayer films by connecting macroscale synthesis parameters to the mesoscale morphology.

In a KMC–CFD coupling study, Crose et al. [215] investigated the Si CVD process. The material concentrations, temperature, and pressure calculated by the CFD method were used in KMC calculations, and then the mass and energy transfer information calculated by the KMC method can be used in the CFD method.

### 5.4. Nano-Meso-Macro Scale

In terms of nano-meso-macro scale coupling, due to the theoretical difficulties there are few relevant studies. A typical example is the MD–PF–CFD coupling. Momeni et al. [216] investigated the CVD preparation of 2D WSe_2_ materials. The heat and flow distributions in the reactor and the precursor concentration distributions were first calculated by CFD simulations. Then, the edge energies of the film were calculated by MD simulations. Finally, the precursor concentrations and direction-dependent edge energies calculated before were transferred to the PF model to predict the morphology and coverage of the grown 2D WSe_2_ material.

**Figure 7:**
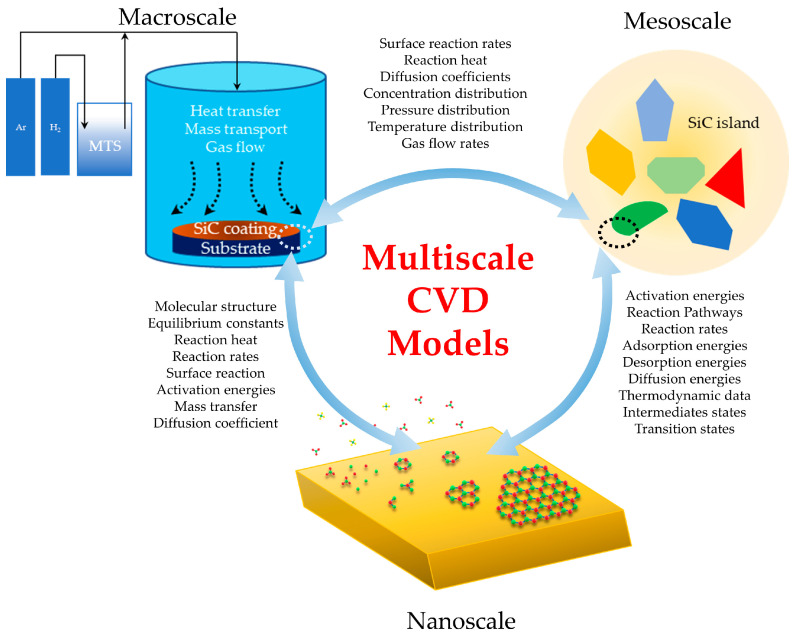
Schematic diagram of information transfer between different scales, aimed at developing the multiscale coupling CVD simulation models.

Based on the discussion above, it can be found that the information transfer between different scales is the key problem in developing the multiscale coupling models, which are summarized in a prospective view in Figure 7. The parameter used in one scale can be calculated more accurately in another scale, which is a common way to establish a multiscale coupling model. Moreover, the multiscale coupling CVD model can be established by a variational method under different scales, which is still a challenge to work through in the future.

## 6. Conclusions

Multiscale modeling has become an important tool in the study and optimization of the CVD process. By bridging the gap between different scales, multiscale models provide a comprehensive understanding of the complexity between various parameters that influence the quality and efficiency of CVD-deposited films and coatings.

This review has explored the critical aspects of multiscale CVD modeling, beginning with an overview of the CVD process mechanisms and the key parameters. Various types of models at different scales have been summarized, including nanoscopic models that capture atomic or molecular interactions, mesoscopic models that describe surface phenomena and nucleation, and macroscopic models that simulate fluid dynamics, heat transfer, and mass transport within the reactor. Each of these models offers unique insights that contribute to the overall understanding of the CVD process, and when integrated into a multiscale framework, they enable the prediction and optimization of deposition outcomes with greater accuracy.

Also, typical multiscale coupling CVD models have been highlighted to illustrate their practical applications and benefits. These models are not only essential for the design and scale-up of CVD reactors but also for the development of advanced materials with excellent properties. By capturing the detailed mechanisms at the nanoscopic level and connecting them to the larger-scale processes, multiscale models provide a pathway for improving the precision and efficiency of CVD technologies, which is still a challenge to work through in the future.

## Data Availability

Not applicable.
